# Individual Characteristics, Family Factors, and Classroom Experiences as Predictors of Low-Income Kindergarteners’ Social Skills

**DOI:** 10.5539/jedp.v6n1p59

**Published:** 2016-01-12

**Authors:** Shayl Griffith, David Arnold, Mary-Ellen Voegler-Lee, Janis Kupersmidt

**Affiliations:** 1 Department of Psychological and Brain Sciences, Univesity of Massachusetts Amherst, Amherst, MA, USA; 2 Department of Psychology, University of North Carolina, Chapel Hill, NC, USA; 3 Innovation Research & Training, Durham, NC, USA

**Keywords:** social skills, transition to kindergarten, low-income, environmental contexts, ecological perspective

## Abstract

There has been increasing awareness of the need for research and theory to take into account the intersection of individual characteristics and environmental contexts when examining predictors of child outcomes. The present longitudinal, multi-informant study examined the cumulative and interacting contributions of child characteristics (language skills, inattention/hyperactivity, and aggression) and preschool and family contextual factors in predicting kindergarten social skills in 389 low-income preschool children. Child characteristics and classroom factors, but not family factors, predicted teacher-rated kindergarten social skills, while child characteristics alone predicted change in teacher-rated social skills from preschool to kindergarten. Child characteristics and family factors, but not classroom factors, predicted parent-rated kindergarten social skills. Family factors alone predicted change in parent-rated social skills from preschool to kindergarten. Individual child characteristics did not interact with family or classroom factors in predicting parent- or teacher-rated social skills, and support was therefore found for an incremental, rather than an interactive, predictive model of social skills. The findings underscore the importance of assessing outcomes in more than one context, and of considering the impact of both individual and environmental contextual factors on children’s developing social skills when designing targeted intervention programs to prepare children for kindergarten.

## 1. Introduction

Social skills are an important component of school readiness for young children preparing to enter formal schooling, and children’s ability to succeed socially predicts later academic, social and emotional functioning (Belsky & MacKinnon, 1994; Rimm-Kaufman & Pianta, 2000; Wildenger & McIntyre, 2012). For children from low-income backgrounds, who are disproportionately at risk for a wide variety of negative outcomes, social skills play an important protective role (Bulotsky-Shearer et al., 2012; Cohen, 2011; Nix, Bierman, Domitrovich, & Gill, 2013). Low-income children with stronger social skills are perceived by teachers to have better academic ability (Baker, Tichovolsky, Kupersmidt, Voegler-Lee, & Arnold, 2014), are less vulnerable to academic disengagement (Bierman et al., 2010; Fantuzzo & McWayne, 2002), and are better able to navigate learning in a classroom of peers (Coolahan, 2000; Elias & Haynes, 2008). While children from low-income communities typically have social skills that are less developed than their higher-income counterparts (Burchinal et al., 2013), this is not universally the case. Identifying individual and contextual factors that promote positive outcomes despite increased risk is essential for a strengths-based understanding of social skills development among low-income children.

While the literature on disruptive behavior problems in at-risk children is abundant, there has been comparatively less focus on positive social skills that allow for the formation of positive relationships with parents, teachers, siblings, and peers. Critical social skills include the ability to communicate effectively, to follow directions, to cooperate, to be attentive, to appropriately ask for and receive help, and to get along well with other children. These skills are important to school success, independent of children’s academic skills (Rimm-Kaufman, Pianta, & Cox, 2000). Examining social skills as a separate construct from behavior problems also allows evaluation of the predictors of children’s social skills taking into account the effect of behavior problems, which may be especially important for understanding social skill development in children with behavior problems.

### 1.1 Classroom, Family, and Individual Factors, and Children’s Social Skills

Extensive existing research on contextual predictors of social skills development in young children from low- and high-income backgrounds has identified *early classroom and home contexts* as particularly important for social skills development (e.g., Deater-Deckard, Dodge, Bates, & Petit, 1998; Howes, 2000; Pianta & Stuhlman, 2004; Pullataz, 1987). For example, the quality of preschool teachers’ interactions with young children, and subsequent teacher-child relationships, are predictive of later social skills (e.g., Brock & Curby, 2014; Brown, Odom, & McConnell, 2008; Howes, 2000; Howes, Matheson, & Hamilton, 1994; Pianta & Stuhlman, 2004). Preschool teachers’ use of developmentally appropriate practices, which are child-focused, engage children in active learning, and emphasize socioemotional as well as academic development, has also been linked to stronger social skills (Bradekamp & Copple, 1997; Hart et al., 1998; Hirsh-Pasek, Golinkoff, Berk, & Singer, 2009; Jones & Gullo, 1999; Marcon, 1999; Stipek, Feiler, Daniels, & Milburn, 1995).

Research on the linkages between parent-child and child-peer relationships indicates that warm and sensitive parenting is related to better social skills (e.g., Clark & Ladd, 2001; NICHD Early Childcare Research Network, 2004, 2003; Pullataz, 1987). Family stability and lower levels of family stress also predict social skills. For example, children of families that are experiencing more instability related to multiple home moves are rated as less socially skilled (Deater-Deckard et al., 1998; Sidebotham, 2001), as disruption to children’s social networks, and the constant need to reacclimatize to a new home and school environment, may interfere with social skills development (Hoglund & Leadbetter, 2005).

Research further suggests that *individual child factors* predict early social skills, such that competencies in other areas affect the ease with which children develop appropriate social skills (e.g., Hoza et al., 2005; Mendez, Fantuzzo, & Cichetti, 2002). Children with strong language skills have more opportunities for productive social interaction with peers, and competent communicators are more likely to gain social acceptance (Mendez et al., 2002; Odom et al., 2006). Conversely, children with language impairments often have difficulty with social interaction with peers (Hart, Fujiki, Brinton, & Hart, 2004; Marton, Abramoff, & Rosenzweig, 2005). Children who are behaviorally competent are also more likely to have successful peer relationships and develop better social skills, because attention problems limit the ability to acquire social skills through observational learning, and may impede the ability to interpret social cues (Andrade et al., 2012; Semrud-Clikeman, 2010). Hyperactivity may also result in irritating behaviors with peers, preventing opportunities for social skill development through positive interactions (Nijmeijer et al., 2008). Aggressive or hostile behaviors may be threatening to other children, resulting in peer rejection, which may further impede the development of social skills (Makami & Hinshaw, 2003; Nijmeijer et al., 2008). These variables are of particular interest because they have direct implications for children’s immediate social interactions, which over time, presumably affect the development of their social skills.

### 1.2 An Ecological Perspective on Young Children’s Social Skills

Ecological-transactional perspectives on child development emphasize the importance of considering the intersecting roles of both contextual and child-level factors in predicting outcomes (e.g., Bronfenbrenner, 1997; Bronfenbrenner & Ceci, 1994; Cichetti & Lynch, 1994; Sameroff, 1975). This is especially true when studying resilience in at-risk populations, as related contextual and individual risk and protective factors work together in complex ways to influence outcomes. Despite this recognition, relatively few studies of young children’s social skills have examined predictors in multiple contextual domains simultaneously, and even fewer have included individual child characteristics in the same model. When contextual and individual effects are examined in separate analyses, we cannot examine if individual factors predict children’s social skill development over and above the effects of contextual processes, or conversely, whether individual characteristics may strengthen or weaken the effects of family or classroom contextual factors. Examining different domains of predictors together, rather than separately, provides valuable information on how these factors can work together to contribute to child outcomes (Deater-Deckard et al., 1998; Greenberg et al., 1999).

Deater-Deckard et al. (1998) suggest that when variables from multiple domains are related to an outcome, and to each other, several possibilities exist. First, some domains could be redundant, with their relation to the outcome being accounted for solely by that domain’s inter-relation with another domain. Second, multiple domains could contribute incrementally to the prediction of the outcome, such that factors in that domain contribute to predicting the outcome over and above the contribution of other domains. Third, factors in different domains may interact in predicting the outcome. For example, individual child characteristics could moderate the relations between contextual factors and outcomes.

Studies have examined the relative contributions of home and school factors in predicting young children’s social skills, though we could find none that included individual child characteristics as a predictive domain. Results have yielded mixed conclusions about the relative importance of factors in the family and school domains, even when social skills are rated in the same context (i.e., by teacher vs. parent raters). Vesely, Brown, and Mahaytma (2013) found that both child care sensitivity and maternal sensitivity were related to teacher-rated social outcomes in a low-income sample. Connell and Prinz (2002) found that both parent-child interaction quality and length of time in child-care were predictors of low-income minority kindergarteners’ teacher-rated social skills. Large-scale research conducted by The National Institute of Child Health and Human Development (NICHD) Early Child Care Research Network (2003), which investigated early home, child-care, and classroom predictors of children’s functioning in a mixed income sample, also found that both child care variables (classroom quality and instructional support) and family variables (maternal sensitivity and depression) contributed incrementally to the prediction of teacher-rated social skills. However, this study found that only family variables uniquely predicted children’s mother-rated first grade social skills. Hoglund and Leadbeater (2004) found that, while home factors (household moves and mother’s education) and school factors (classroom levels of victimization and prosocial skills, and school disadvantage) were both related to first grader’s teacher-rated social skills, when these contextual variables were entered into a regression model together, only the classroom factors remained significant predictors. Likewise, McWayne, Cheung, Wright, and Hahs-Vaughn (2012) found that classroom but not family factors uniquely predicted teacher-rated social skills in a low-income sample.

Together, these findings underscore the importance of careful examination of ways that contextual variables intersect to predict children’s social skills, as well as the importance of examining reports of social skills from multiple informants, since patterns of prediction have been shown to differ depending on the informant (e.g., parent vs. teacher; NICHD Early Childcare Research Network, 2003). This is consistent with research on multi-rater congruence for social skills, which has found that inter-rater correlations are much higher between informants who encountered the child in the same setting (e.g., mother/father or teacher/teacher) than between informants in different settings (e.g., parent/teacher or teacher/mental health worker; Achenbach, McConaughy, & Howell, 1987; Fagan & Fantuzzo, 1999). Parents and teachers observe children in different contexts, in which child behaviors may differ, and in which the skills needed to be successful in that context differ (Murray, Ruble, Willis, & Malloy, 2009). Teachers may also rely more heavily on comparison with like-aged peers for rating children’s social skills, and view children more frequently in interaction with non-sibling peers, while parents have fewer opportunities for such comparisons (Diamond & Squires, 1993; Fagan & Fantuzzo, 1999). Cultural differences between parents and teachers in expectations for children’s behavior may also lead to differing perceptions (e.g., Winetsky, 1978).

Examination of reports from multiple informants, rating social skills displayed in multiple contexts, is therefore essential for a comprehensive understanding of social skills development, as each provides unique information about a child’s social functioning (Tassé & Lecavalier, 2000). Multi-informant ratings can also help to address problems with method variance, and may provide convergent evidence. To our knowledge only one other study, the NICHD Early Childhood Network Research, included ratings from both parents and teachers when examining home and school predictors of social skills. However, this mixed income sample had high rates of selective attrition from low income families (e.g., Hamre & Painta, 2005) and did not include individual-level predictors.

### 1.3 Ecological Models of Children’s Behavior Problems

While we did not locate any studies of low income children’s positive social skills that examined the cumulative contributions of individual child characteristics and home and school contextual factors, studies of children’s behavior problems and other negative adjustment outcomes have found that individual and contextual factors in multiple domains contribute incrementally to predicting behavior problems (Deater-Deckard et al., 1998; Greenberg et al., 1999; Vanderbilt-Adriance et al., 2015). Vanderbilt-Adriance et al. (2015) found, for example, that child (inhibitory control and attention), family, and community factors made small but significant incremental contributions to the prediction of parent-rated child conduct problems. Deater-Deckard et al. (1998) found that child factors (temperament and medical problems), and sociocultural, parenting, and peer-related domains of risk added incrementally to the prediction of externalizing behavior problems, measured by combined ratings from teachers, parents, and peers. Consistent with ecological theory, results of these studies suggest that examination of individual child factors, in addition to contextual predictors, is important for predicting outcomes in at risk populations.

In accordance with the third multivariate model proposed by Deater-Deckard et al. (1998), it is also possible that individual factors have a moderating effect on the relation between contextual predictors and outcomes, such that context affects outcomes differently for different children (Bronfenbrenner, 1977). For example, studies have found that negative emotionality moderated the relationship between family risk factors, including maternal depression, parent-child conflict, and household overcrowding, and children’s social competence (Bush, Lengua, & Colder, 2010; Chang, Cheong, & Shaw, 2012; Lengua, 2002). No studies have included individual characteristics, such as language skills, attention/hyperactivity, and aggression, along with home and school factors when examining predictors of young children’s social skills. As such, it is not known whether these child characteristics are empirically redundant with contextual predictors, add incrementally to the prediction of social skills, or interact to change the relationships between contextual factors and social skills.

### 1.4 The Present Study

In the present study, we examine from an ecological perspective the intersecting contributions of preschool child characteristics and classroom and family contextual factors in predicting kindergarten social skills at home and at school in a low-income population. We first examine a cumulative effects model, where preschool family, classroom, and individual factors were hypothesized to each contribute incrementally to predicting kindergarten social skills. We then examine an interactive model, where individual factors were hypothesized to moderate the effects of family and classroom factors on child social skills. Preschool classroom, family, and child variables were expected to predict both final kindergarten social skills, as rated by parents and teachers, and the change in social skills from preschool to kindergarten. Specifically, we hypothesized that preschoolers’ language skills, lower inattention/hyperactivity, and lower aggression would predict better social skills; that preschool teachers’ warmth and sensitivity, and use of developmentally appropriate teaching practices, would predict better social skills; and that warmer parenting, and greater housing stability would predict better social skills. We further hypothesized that individual child characteristics (language skills, inattention, and aggression) would each moderate the relationship between (a) family factors and classroom factors and children’s parent- and teacher-rated social skills, and (b) classroom factors and children’s parent- and teacher-rated social skills in kindergarten. It was expected that better language skills and behavioral competence would buffer the negative effects of housing instability, harsh parenting, less teacher warmth, and developmentally inappropriate practices on social skills.

This study adds to existing literature in a number of ways. First, the inclusion of individual child factors (language ability, inattention/hyperactivity, and aggression) in addition to family and classroom variables may illuminate important patterns in the relationships between individual and contextual predictors and social skills. Second, including outcome ratings from multiple informants allows examination of predictors of social outcomes in different settings (home and school), where behavior and behavioral demands may differ. Third, the longitudinal design adds to a literature that is largely cross-sectional. Finally, the present study examines the predictors of social skills in a low-income and ethnically diverse sample, thus adding to the smaller existing knowledge base about social skills development in this particularly vulnerable population.

## 2. Method

### 2.1 Participants

Participants were 389 children (199 boys; 190 girls) attending 68 Head Start and Community Child Care (CCC) preschool centers in rural, suburban, and urban counties in North Carolina. Children were recruited in the fall of their final preschool year (*M* = 56.08 months, *SD* = 3.71). Half of the children in the sample were identified by their families as African-American/Black (50.6%), 37.8% as White, 3.6% as Hispanic, 3.3% as bi-racial (Black/White), 1.3% as Asian, and another 3.1% as having other bi- or multi-racial identities. At the time of study enrollment, 37.8% of families reported receiving at least one form of public assistance, including food stamps/Temporary Assistance for Needy Families (TANF), the Special Supplemental Nutrition Program for Women, Infants, and Children (WIC), or other welfare assistance. Data were collected as part of an intervention study for the Building Bridges program, which was designed to increase kindergarten readiness by accelerating the development of language and emergent literacy, mathematics, and behavioral skills in preschoolers. This program was designed to be implemented by teachers, and the Building Bridges program provided implementation training to preschool teachers in one of three conditions: (1) Workshop Plus, which provided training, as well as ongoing consultation, (2) Workshop only, which provided training only, and (3) Control. This study used pre-test data from the pre-K year, as well as kindergarten follow-up data. Kindergarten teachers were not included in the intervention program. The intervention did not have a substantial effect on social skills (Kupersmidt et al., 2010). Children who had data for at least one predictor and one outcome variable for the present study were included in the sample.

Head Start centers were first recruited for participation, and then CCC centers were identified to match the geographic location and other general characteristics of the Head Start centers. CCC centers were eligible to participate if they had at least one classroom in which 50% or more of the children were 4-years-old, and if 50% or more of their students were designated as low-income students. The present study utilizes data collected from 120 preschool classrooms (58 Head Start; 62 CCC) in 68 preschool centers (23 Head Start; 45 CCC). In total, 1,997 children from these centers were invited to participate and, of those, 1,004 were given permission to participate by parents. 79 children did not meet criteria for participation, and were excluded from the sample. Children were not eligible to participate if they had disability that did not allow participation in direct assessments for children, if English was not their primary language, if they were not enrolled in the center at the time of data collection, or if they only attended the center in the afternoon.

### 2.2 Procedure

Once the directors of the targeted centers consented to participate in the study, presentations were made to preschool classroom teachers to invite their participation. Consenting preschool teachers were interviewed individually by research staff in the fall of the pre-K year. As part of this interview, teachers rated the social skills and disruptive behavior problems of each child in their classroom, and filled out questionnaires about their teaching beliefs and practices. Preschool teachers were also observed in their classrooms by project staff and rated on their interactions with the children. Kindergarten teachers of the children in the present sample were approached in the fall of the child’s kindergarten year and were asked to similarly report on the children’s social skills and disruptive behaviors. Teachers received $15 for completing each interview.

Children in the preschool classrooms of participating teachers were given letters describing the study and consent forms to take home to their parents. Children whose parents gave consent participated in a 30–45 minute language and academic skills assessment in the fall of the pre-K year, conducted by research staff in a private setting at the child’s preschool. Children received a book and stickers for their participation in the assessment. Consenting parents filled out and returned questionnaire packets in the fall of their child’s pre-K year, and again during the kindergarten year. Parents received a $25 gift card for completing the questionnaire packet at each time point.

### 2.3 Measures

#### 2.3.1 Social Skills

Parents and teachers completed their respective versions of the *Social Skills Rating System* (SSRS; Gresham & Elliott, 1990) in the fall of the pre-K year, and again in the fall of the kindergarten year. The SSRS assesses children’s positive social skills, using a 30-item scale for teachers, and a 39-item scale for parents. Parents and teachers rated the frequency with which children displayed positive social behaviors at home and at school, respectively (e.g., “makes friends easily” and “volunteers to help peers with classroom tasks”) on a 3-point Likert scale (“never”, “sometimes”, or “often”). The SSRS is a widely used scale that has been found to be reliable and valid for use with preschoolers, with children of different ethnicities, and with children from both clinical and non-clinical populations (Fantuzzo, Manz, & McDermott, 1998; Van der Oord et al., 2005; Walthall, Konold, & Pianta, 2005). Scores from all items on each form were averaged to obtain overall parent-rated and teacher-rated social skills scores, on a scale from 0–2, with higher scores indicating greater social skill. Cronbach’s alpha was .94 for the teacher ratings, and .90 for the parent ratings.

#### 2.3.2 Teacher Warmth and Sensitivity

Teachers were observed in their classroom by trained research staff, who rated the quality of teachers’ interactions with students the *Caregiver Interaction Scale* (CIS; Arnett, 1989). Ratings were completed in vivo, rather than from video recordings. Observers rated teachers on 26 items, assessing the extent to which they displayed sensitivity (e.g., “speaks warmly to the children”), detachment (e.g., “doesn’ t seem interested in children’s activities”), permissiveness (e.g., does not supervise the children very closely), and harshness (e.g., “speaks with irritation or hostility to the children”) in their interactions with children. For each item, teachers were rated on a 4-point Likert scale from 1 (“not at all”) to 4 (“very much”). This scale has demonstrated good inter-rater reliability, with coefficients ranging from .75 to .97 (Jaeger & Funk, 2001). Scores for the 26 items were reverse scored where appropriate, and averaged to form an overall score for warmth and sensitivity, with a higher score indicating a warmer and more sensitive interaction style. Cronbach’s alpha for the 26-item scale in this sample was .91.

#### 2.3.3 Developmentally Appropriate Teaching Practices

Developmentally appropriate teaching practices were measured with the *Pre-K Teacher Beliefs and Practices Scale* (Marcon, 1999). The scale consists of seven items assessing teachers’ beliefs, and a corresponding seven items assessing teachers’ practices concerning the developmental goals of the classroom, the role of the teacher, and the understanding of how children learn best. Teachers were asked to respond to each item (e.g., “I believe that my role as a teacher of preschool children is to dispense knowledge” vs. “facilitate learning”) on a scale from one to ten of opposing beliefs or practices. Scores for all 14 items were averaged to create an overall score for teachers’ beliefs and practices, with higher scores indicating more child-initiated, socioemotionally-focused classrooms. This scale has been validated for use with preschool programs, including Head Start and public pre-kindergarten programs, serving low income, primarily African-American populations (Marcon, 1999). Cronbach’s alpha for the overall 14-item scale in this sample was .81.

#### 2.3.4 Parenting Warmth

Parenting warmth was measured using 4 items from the *Child Rearing Practices Report* (CRPR; Block, 1965). Parents rated the extent to which each item (e.g., “My child and I have warm, intimate moments together”) was true for them on a five-point Likert scale from 1 (“exactly”) to 5 (“not at all”). An overall score was formed by reverse scoring and taking the average of all item scores, so that a higher score indicated warmer parenting. This questionnaire has good construct validity and has been used with families with children of different ages, socioeconomic levels, and national origins (Block, 1965; Block & Christiansen, 1966). Cronbach’s alpha for the 4-item scale in this sample was .65.

#### 2.3.5 Housing Instability

Parents were asked to report the number of household moves that had taken place since the child’s birth.

#### 2.3.6 Inattention/Hyperactivity

Preschool teachers reported on inattentive/hyperactive behaviors of all children in their classrooms using the Inattention/Hyperactivity subscale of the *IOWA Conners Teachers Rating Scale* (Conners; Loney & Milich, 1982). The subscale consists of 5 items, and teachers rated how characteristic each behavior (e.g., “fidgets”) is for a child on a 4-point Likert scale from 0 “not at all” to 3 “very much”. The 5 items were averaged to give each child an inattention/hyperactivity score. The Conners has been widely validated for use with children (e.g., Casat, Norton, & Boyle-Whitesel, 1999; Pelham et al., 1989), and construct equivalence has been demonstrated with children from different ethnic groups (Reid, Casat, Norton, Anastopoulos, & Temple, 2001). Cronbach’s alpha for the 5-item scale in this sample was .88.

#### 2.3.7 Aggression

Parents reported on their children’s aggressive behaviors using the Types Of Aggression (TOA) Rating Scale (Kupersmidt, Bryant, & Willoughby, 2000). The scale consists of 12 items on two subscales measuring overt and covert aggression. Parents rated the frequency of aggressive behaviors on a 5-point Likert scale (1 = Once a month or less; 5 = Many times a day). Scores for the 12 items were averaged to create an overall aggression score. This measure has been demonstrated to exhibit acceptable test–retest and inter-rater reliability, as well as demonstrated criterion validity for general measures of conduct problems, hyperactivity, and adult and peer conflict in the classroom setting (Willoughby, Kupersmidt, & Bryant, 2001). The internal consistency of the total aggression score in the sample was good (α = .91).

#### 2.3.8 Language Ability

Children’s language ability was measured with the Peabody Picture Vocabulary Test, Third Edition (PPVT-III; Dunn & Dunn, 1997), which is a normed and extensively validated test of receptive vocabulary. This test requires children to respond to words by selecting the correctly corresponding picture from a group of four pictures. Standardized scores for each child on the PPVT-III were used as the measure of language ability in this study. Split-half reliability of the PPVT-III has been reported at .80 (Dunn & Dunn, 1981). The measure has also been found to be valid for use with children from diverse ethnic and socioeconomic backgrounds (e.g., Campbell, Bell, & Keith, 2001; Washington & Craig, 1999).

### 2.4 Data Analytic Plan

The 389 children were recruited from 120 preschool classrooms. The number of children per preschool classroom ranged from one to seven, with a significant number containing only one child per classroom. At the kindergarten level, the 238 children with kindergarten teacher data were from 103 classrooms, and again the number of children per kindergarten classroom ranged from one to seven (*M* = 2.31, *SD* = 1.28), with 32% of classrooms having one study child. Given the very small amount of nesting in this sample, and given concerns about using hierarchical linear modeling when the average number of observations per group is less than five (see Clarke, 2008; Clarke & Wheaton, 2007; Theall et al., 2011), when data are unbalanced (unequal group sizes; Clarke, 2008), and when there is a large proportion of singletons (Bell, Morgan, Kromney, & Ferron, 2010), hierarchical regression models, which provide unbiased regression weight estimates, were used to predict children’s kindergarten social skills scores from preschool individual, classroom, and family factors (e.g., Bush, Lengua, & Colder, 2010).

Hierarchical regression models were run separately for teacher- and parent- rated social skills. Control variables (child sex, age, ethnicity, preschool type, whether the child’s family was receiving public assistance, and intervention status) were entered first into the overall regression model, followed by classroom factors (teacher warmth and developmentally appropriate practices), family factors (parenting warmth and housing instability), and child characteristics (language ability, inattention/hyperactivity, aggression). Following the procedure by Deater-Deckard et al., 1998, a series of hierarchical regression models were then run where each of the domains (child, classroom, and family) were entered last in the model to obtain the unique change in R^2^ for that domain. Although the intervention program did not show effects on social skills in the preschool year, the possibility of delayed effects underscores the importance of continuing to control for intervention status in these regression models.

Each regression model was run both with and without controlling for preschool social skills. The initial model (Model 1) controlled for sex, age, ethnicity, public assistance, preschool center type, and intervention status. The second model (Model 2) additionally controlled for pre-K parent- or teacher-rated social skills. Arguments could be made for or against controlling for initial social skills, particularly given that the family and child variables could be expected to have longer term influence on social skills, while children were exposed to pre-K classroom variables only for that year. We believe that omitting initial skills likely overestimates the effects of some predictor variables, while including them probably “overcorrects”, likely underestimating the importance of predictors with continuing effects. Certainly controlling for initial skills provides a very rigorous test of predictor importance, given that all prior influences on skills have been controlled. Given this dilemma, presenting both models seemed optimal.

To evaluate whether child variables moderated the effects of the home and school context, we ran another series of hierarchical regressions. Interactions of child variables with classroom and family variables were examined separately for parent- and teacher- rated social skills, controlling for pre-K social skills. The interaction terms were entered in pairs in separate regression models using forced entry, after adding control, classroom, family, and individual variables. Each of the three child variables were entered with (a) the two family variables, and then (b) the two classroom variables. For example, the *Language Ability x Parenting Warmth* and *Language Ability x Housing Instability* interaction terms were entered together, resulting in 6 regression models. We examined the significance of coefficients for each interaction term, as well as the R^2^ change for the pair of terms.

Missing data were minimal for all variables except the kindergarten teacher-rated social skills variable, where the missing data were moderate. For this variable, 238 of the 389 children had data. Missing data at pretest for the teacher-rated social skills variable was small, with 374 of 389 children having complete data. The increased percentage of missing data from pre-K to kindergarten resulted from kindergarten teachers of students in the study who could not be contacted, or did not agree to fill out rating forms for the children when they entered kindergarten. Results of t-tests run for each of the seven predictor variables and parent-rated social skills suggest that children in the sample who had complete kindergarten teacher data were not significantly different from those who did not with respect to these variables. Multiple imputation using SPSS 22.0 was used to account for the missing data in all predictor and outcome variables, and the regression analyses were run on 10 imputed datasets. Teacher and parent social skills ratings on the SSRS, collected in the spring of the preschool year, were added as auxiliary variables to improve prediction (Collins, Schafer, & Cam, 2001; Raykov & Marcoulides, 2014). Interaction terms were created before being added to the imputation model, to obtain unbiased estimates of the interaction coefficients (Von Hippel, 2009). Results pooled from the imputed datasets are reported below. Results from the imputed data set did not differ appreciably in relations between variables or effect size from the results run without the imputed data.

## 3. Results

### 3.1 Descriptive Statistics

Table 1 presents means, standard deviations, and intercorrelations for study variables. Means were consistent with those expected for a low-income community sample. Preschool teacher-rated social skills were correlated positively with teacher warmth, developmentally appropriate teaching practices, and language ability, and negatively with inattention/hyperactivity and housing instability. Preschool parent-rated social skills were positively correlated with parenting warmth and language ability, and negatively correlated with inattention/hyperactivity and aggression. At the preschool level, the correlation between teacher- and parent- rated social skills was small but significant (*r* = .12), while at the kindergarten level, the correlation between teacher- and parent- rated social skills was medium (*r* = .29). There was a medium correlation between preschool and kindergarten teachers’ social skill ratings (*r* = .34), while the correlation between parent ratings at preschool and kindergarten was large (*r* = .66).

### 3.2 Predictors of Parent- and Teacher-Rated Social Skills

#### 3.2.1 Teacher-Rated Kindergarten Social Skills

Developmentally appropriate teaching practices, language ability, inattention/hyperactivity, and aggression each predicted teacher-rated social skills in Model 1, controlling for all other predictor variables. Language ability, inattention/hyperactivity, and aggression predicted teacher rated social skills, controlling for pre-K teacher-rated social skills in Model 2, while developmentally appropriate teaching practices was a marginally significant predictor. All regression weights were in the hypothesized direction (Table 2). When each domain was entered last into the regression model, classroom variables and child characteristics each incrementally improved prediction of kindergarten teacher rated social skills in Model 1, while only child characteristics added uniquely to the prediction of social skills in Model 2. The classroom domain was a marginal predictor of social skills in Model 2 (*p* = .06). Model 1 accounted for 33% of the variance in kindergarten teacher-rated social skills, and Model 2 accounted for 35% of the variance in kindergarten teacher-rated social skills.

#### 3.2.2 Parent-Rated Kindergarten Social Skills

Preschool-rated parenting warmth, housing instability, and inattention/hyperactivity predicted kindergarten parent-rated social skills in Model 1, while parenting warmth and housing instability alone predicted parent-rated social skills in Model 2. All regression weights were in the hypothesized direction (Table 3). When each domain was entered last into the regression model, family factors and child characteristics each incrementally improved prediction of kindergarten teacher rated social skills in Model 1, while only family factors added uniquely to the prediction of social skills in Model 2. Model 1 accounted for 25% of the variance in kindergarten parent-rated social skills, and Model 2 accounted for 48% of the variance in kindergarten parent-rated social skills.

#### 3.2.3 Moderating Effect of Child Characteristics on Classroom and Family Variables

There were no significant interactions between child characteristics and classroom and family factors in predicting parent- or teacher-rated social skills. Additionally, none of the *Child x Family* or *Child x Classroom* interaction pairs improved prediction of parent-or-teacher- rated social skills. The interactions were in varying directions.

## 4. Discussion

The present study investigated the ways that individual, classroom, and family factors at preschool age predicted children’s teacher- and parent-rated social skills in kindergarten, by investigating cumulative and interactive models of prediction. Individual characteristics (language, inattention/hyperactivity, and aggression), and classroom factors (developmentally appropriate teaching practices) added incrementally to the prediction of kindergarteners’ overall teacher-rated social skills. Individual characteristics also predicted the change in teacher-rated social skills across the transition from preschool to kindergarten, while the classroom domain was a marginal predictor of this change. Individual characteristics (inattention/hyperactivity) and family factors (parenting warmth and housing instability) added incrementally to the prediction of kindergarten parent-rated social skills, while only family factors predicted the change in parent-rated social skills across the transition to kindergarten.

Individual characteristics did not significantly moderate the relationships between classroom and family factors and teacher-and parent-rated social skills, suggesting the domains add incrementally, rather than interactively, to the prediction of social skills in this population. The present study adds to theory and the research literature by being the first to examine early individual characteristics along with home and school contextual predictors of social skills together in a low-income population. The findings suggest that factors in each domain are important for predicting kindergarteners’ social skills, but may be differentially important in the home verses the classroom.

Children’s individual characteristics in preschool emerged as the strongest predictive domain for kindergarten social skills as rated by teachers, suggesting that children’s language ability, levels of inattention/hyperactivity, and aggression may be particularly important for children’s social skills in a classroom context. This is consistent with research that suggests that children’s ability to communicate well with peers, to attend to social cues and learn from social interactions, and to refrain from irritating or threatening behaviors, affects their ability to develop social skills through positive peer interactions (e.g., Andrade et al., 2012; Hart et al., 2004; Nijmeijer et al., 2008). The classroom is likely to be the context in which non-sibling peer-to-peer interactions for young children are most important and most frequent, and it follows that related skills that facilitate positive interactions will be especially important for social skills development in this context. It is notable that preschool child characteristics in this study predicted the change in social skills from preschool to kindergarten more strongly than did preschool classroom factors, which was only a marginal predictor. This is in contrast to previous studies which have found that classroom characteristics do predict change in teacher-rated social skills (e.g., McWayne et al., 2012; NICHD, 2003). However, these studies did not include individual child characteristics in their models. One possible explanation is that some of the effect of classroom factors on social skills is redundant with the effect of individual child characteristics, such that classroom factors have a stronger relationship with language or behavioral competence than with social skills directly. Another possibility is that characteristics of the preschool classroom, such as teachers’ warmth or teaching practices, may show more subtle effects on children’s social skills that manifest over periods of time longer than a year.

By contrast, family factors most strongly predicted children’s parent-rated social skills, and particularly the change in social skills at home from preschool to kindergarten. Children’s inattention/hyperactivity predicted overall kindergarten parent-rated social skills, and language skills and aggression were not significant predictors. This suggests that children’s experience of warmth and stability in the home might have particular importance for the development of their social skills in the home context, where children are primarily interacting with adult family members and siblings. The findings further suggest that children’s inattentive and hyperactive behaviors in particular are predictive of their ability to meet the social demands of the home environment, more than aggressive behaviors or language ability.

Individual characteristics did not moderate the effects of family and individual characteristics in this sample, again suggesting that family, classroom and individual variables contribute relatively independently to social skills development. Though it is difficult to conclusively establish the absence of interactions, effect sizes were very small as well as non-significant, and interactions were not even consistently in the same direction. While some cross-sectional research suggests that certain child characteristics, such as negative emotionality, moderate the effects of disadvantageous family characteristics on child outcomes (e.g., Bush, Lengua, & Colder, 2010; Chang, Cheong, & Shaw, 2012; Lengua, 2002), it seems clear that, in this sample, individual and contextual factors were additively related to social outcomes one year later, providing support for incremental, rather than interactive, predictive relationships between the contextual and individual domains.

A major strength of this study was the use of multiple raters to report on children’s social skills in two different domains: home and school. The findings suggest that contextual and individual predictors are differentially important for children’s social skills in these different contexts. These results are consistent with research that has found only modest associations between social skills ratings of raters in different contexts. While one explanation could be individual differences in the raters themselves, research has also shown that raters observing children in the same context are highly congruent, while those observing children in different contexts are not (Achenbach, McConaughy, & Howell, 1987; Fagan & Fantuzzo, 1999), suggesting that the behaviors children display, and how those behaviors are perceived, may also differ according to context (e.g., Murray et al., 2009). This underscores the importance of assessing outcomes in more than one context, as studies of social skills that include ratings reflecting functioning in only one context are likely missing a large part of the picture.

Studies including predictors and outcomes rated by the same person are also potentially limited by method variance. In this study, while predictors and outcomes were both rated by parents and teachers, there are several indications that method variance was not the sole cause of the results. First, for kindergarten teacher-rated social skills, the strongest predictors were children’s individual characteristics, which were rated by preschool teachers (inattention/hyperactivity), parents (aggression), and by direct assessment (language ability). Although family factors (reported by parents) were the strongest predictors on parent-rated social skills, child aggression (also rated by parents) was not a significant predictor, while inattention/hyperactivity, which was rated by teachers, was a significant predictor. Including ratings from preschool and kindergarten teachers, parents, and direct assessment measures was a strength of this study, as it provided convergent evidence, and reduced issues of method variance.

The longitudinal design of the study allowed examination of the relationship between predictors in multiple domains and social skill outcomes across the transition to elementary schooling, a time which has been demonstrated to be critical to children’s later school and social success, extending a largely cross-sectional literature. The fact that individual and family factors predicted change in teacher and parent ratings over the preschool-to-kindergarten transition suggests that these factors are all important continuing pieces of the puzzle to consider for social skill development during this period of transition to kindergarten. As a group, the six classroom, family, and individual predictors created a strong model, accounting for one third to one half of the variance in parent- and teacher-rated kindergarten social skills. Programs aimed at improving the social development of children at risk across the transition to kindergarten therefore have many potential targets of intervention, and an approach which encompasses all three contexts-classroom, family, and child - may be especially beneficial.

### 4.1 Limitations

While this study had many strengths, there were also limitations. Firstly, while the sample was recruited from preschools in low income areas, and included only preschools serving a majority of low income students, specific information about socioeconomic status (via family income and parental education) for each family was not available. We were therefore not able to investigate the effect of relative wealth or education of the parents of the low-income children in the sample. However, information was collected about the eligibility and need of the family for public assistance programs. Since eligibility for public assistance programs is largely income-based, this variable was used to provide basic information about the relative income levels of the low-income families in the sample. Secondly, since data were collected as part of a separate study, comprehensive measures of some of the constructs of interest in this study, such as parenting and housing instability, were not available. The four item parenting measure used in this study also evidenced only moderate internal reliability, potentially due in part to the small number of items. Future studies should examine effects of variations in education and wealth within lower income populations on children’s social development, and should use more comprehensive and thorough measures of these family constructs. Thirdly, while the longitudinal nature of the study provided important information about the relationships between contextual factors and social skill over time, and controlled for related variables in the analysis, it is possible that additional important variables were not included. Additionally, the correlational design of this study does not allow for causal conclusions. Future intervention research in this area could inform causal models and provide essential information for translation of research in this area to real-world settings.

### 4.2 Conclusions

The findings of this study, though preliminary, could have important implications for preschool education policies, particularly surrounding screening for future social skill deficits. The results suggest that intervention programs seeking to improve children’s social skills at school and with peers may benefit from a focus on language development as well as impulse and aggression control. Earlier screening for language and behavior problems may also help facilitate the provision of early assistance for children at risk for developing social problems in the pre-kindergarten and early school years. Results further suggest that interventions to improve the warmth and stability of the home environment may also be important for young children’s social skills in that context. In general, then, intervention programs targeting social skill development may benefit from an integrated approach, which includes teachers, parents *and* children as the targets of intervention.

## Figures and Tables

**Table 1:** Descriptive statistics for study variables

Variable	1.	2.	3.	4.	5.	6.	7.	8.	9.	10.	11.	12.	13.	14.	15.
1. Child Age															
2. Sex	.01														
3. Ethnicity	.09	−.09													
4. CCC	−.11*	−.01	−.55**												
5. Public Assistance	.04	−.02	.39**	−.46**											
6. Teacher-Rated Social Skills (Pre-K)	.07	−.14**	−.15**	.10*	−.08										
7. Parent-Rated Social Skills (Pre-K) Classroom Factors	.10	−.22**	−.03	.22**	−.16**	.12*									
8. Teacher Warmth and Sensitivity	−.02	−.02	−.07	−.05	−.04	.14**	−.06								
9. DA Teaching Practices Family Factors	−.07	.06	−.13**	−.01	−.03	.17**	−.13*	.18**							
10. Parenting Warmth	−.03	−.12*	−.03	.10	−.01	.05	.40**	−.09	−.09						
11. Housing Instability Child Characteristics	.02	−.09	.16**	−.22**	.23**	−.12*	−.09	−.08	−.06	−.02					
12. Language Ability	.01	.01	−.46**	.43**	−.33**	.24**	.17**	.09	.05	.05	−.13*				
13. Inattention/Hyperactivity	−.07	.20**	.06	−.03	.00	−.56**	−.15**	−.11*	−.05	−.04	.00	−.12*			
14. Aggression Outcomes (Kindergarten)	−.06	.13*	−.08	−.06	.07	−.06	−.25**	.05	−.01	−.15**	.07	.07	.20**		
15. Teacher Rated Social Skills	.12	−.32**	−.02	.09	−.09	.34**	.30**	−.01	.11	.15*	.01	.21**	−.37**	−.27**	
16. Parent-Rated Social Skills	.04	−.14**	−.14**	.24**	−.16**	.14**	.66**	−.04	−.07	.37**	−.17**	.15**	−.16**	−.20**	.29**
Mean	56.08	.51	.62	.57	.38	1.46	1.34	3.23	6.99	4.15	1.59	94.90	.82	1.26	1.40
SD	3.71	.50	.49	.50	.49	.27	.26	.63	.93	.54	1.74	15.86	.68	.44	.39

*Note*. For sex, girls = 0, boys = 1. For ethnicity, European-American = 0, Minority = 1. For CCC, Head Start = 0, CCC = 1. For receiving public assistance, no = 0, yes = 1. PPVT-III = Peabody Picture Vocabulary Test. DA = Developmentally Appropriate.

* *p* < .05,

** *p* < .01,

*** *p* < .001.

**Table 2:** Hierarchical regression analysis predicting kindergarten teacher-rated social skills, with and without controlling for preschool social skills

	Model 1			Model 2		
	
	*B*	*SE*	Δ R^2^	*B*	*SE*	Δ R^2^
Control			.14***			.24***
Child Age	.11†	.06		.10	.06	
Sex	−.24***	.05		−.24***	.06	
Ethnicity	.11	.08		.11	.08	
CCC	.01	.07		.01	.07	
Public Assistance	−.06	.08		−.06	.08	
WO	−.05	.08		−.05	.08	
WP	.06	.07		.07	.07	
Pre-K Teacher SSRS				.14†	.07	
Classroom Factors			.02*			.02†
CIS	−.07	.06		−.07	.06	
DA Teaching	.14*	.06		.12†	.06	
Family Factors			.01			.01
Parenting Warmth	.09	.07		.09	.07	
Housing Instability	.04	.06		.05	.06	
Child Characteristics			.15***			.08***
Language Ability (PPVT-III)	.20*	.08		.18*	.07	
Aggression	−.15*	.07		−.16*	.06	
Inattention/Hyperactivity	−.27***	.06		−.20*	.08	
R^2^ Model total	.33***			.35***		

*Note*. For sex, girls = 0, boys = 1. For ethnicity, European-American = 0, Minority = 1. For CCC, Head Start = 0, CCC = 1. For receiving public assistance, no = 0, yes = 1. PPVT-III = Peabody Picture Vocabulary Test. DA = Developmentally Appropriate. Regression weights are standardized. Regression weights are from the full model including all predictors. Δ R^2^ for the classroom, family and child domains reflects the change in R^2^ when that domain is entered last in the model.

* *p* < .05,

** *p* < .01,

*** *p* < .001

† *p* < .10

**Table 3:** Hierarchical regression analysis predicting kindergarten parent-rated social skills, with and without controlling for preschool social skills

	Model 1			Model 2		
	
	*B*	*SE*	Δ R^2^	*B*	*SE*	Δ R^2^
Control			.09***			.45***
Child Age	.06	.05		.004	.04	
Sex	−.07	.05		.008	.04	
Ethnicity	−.01	.06		−.08	.05	
CCC	.13*	.06		.05	.06	
Public Assistance	−.04	.06		.006	.05	
WO	−.02	.06		−.02	.05	
WP	.06	.06		.04	.05	
Pre-K Teacher SSRS				.58***	.05	
Classroom Factors			.004			.000
CIS	−.03	.05		−.02	.04	
DA Teaching	−.05	.05		.00	.04	
Family Factors			.11***			.02**
Parenting Warmth	.32***	.05		.12**	.05	
Housing Instability	−.11*	.05		−.09*	.04	
Child Characteristics			.03*			.006
Language Ability (PPVT-III)	.03	.06		−.03	.05	
Aggression	−.10†	.06		−.02	.05	
Inattention/Hyperactivity	−.11*	.05		−.07	.04	
R^2^ Model total	.25 ***			.48***		

*Note*. For sex, girls = 0, boys = 1. For ethnicity, European-American = 0, Minority = 1. For CCC, Head Start = 0, CCC = 1. For receiving public assistance, no = 0, yes = 1. PPVT-III = Peabody Picture Vocabulary Test. DA = Developmentally Appropriate. Regression weights are standardized. Regression weights are from the full model including all predictors. Δ R^2^ for the classroom, family and child domains reflects the change in R^2^ when that domain is entered last in the model.

* *p* < .05,

** *p* < .01,

*** *p* < .001

† *p* < .10
